# Dual-amplification system based on CRISPR-Cas12a and horseradish peroxidase-tethered magnetic microspheres for colorimetric detection of microcystin-LR

**DOI:** 10.1007/s00604-023-05887-9

**Published:** 2023-07-20

**Authors:** Pian Wu, Man Zhang, Xiaoting Xue, Ping Ding, Lei Ye

**Affiliations:** 1 Division of Pure and Applied Biochemistry, Department of Chemistry, Lund University, 22100 Lund, Sweden; 2 Xiang Ya School of Public Health, Central South University, Changsha, 410078 Hunan China

**Keywords:** Microcystin-LR, CRISPR-Cas12a, Horseradish peroxidase, Colorimetric detection

## Abstract

A novel dual-amplification system based on CRISPR-Cas12a and horseradish peroxidase (HRP) was developed for colorimetric determination of MC-LR. This dual-amplification was accomplished by combining the nuclease activity of CRISPR-Cas12a with the redox activity of HRP. HRP linked to magnetic beads through an ssDNA (MB-ssDNA-HRP) was used to induce a color change of the 3,3′,5,5′-tetramethylbenzidine (TMB)-H_2_O_2_ chromogenic substrate solution. Specific binding of MC-LR with its aptamer initiated the release of a complementary DNA (cDNA), which was designed to activate the trans-cleavage activity of CRISPR-Cas12a. Upon activation, Cas12a cut the ssDNA linker in MB-ssDNA-HRP, causing a reduction of HRP on the magnetic beads. Consequently, the UV–Vis absorbance of the HRP-catalyzed reaction was decreased. The dual-signal amplification facilitated by CRISPR-Cas12a and HRP enabled the colorimetric detection of MC-LR in the range 0.01 to 50 ng·mL^−1^ with a limit of detection (LOD) of 4.53 pg·mL^−1^. The practicability of the developed colorimetric method was demonstrated by detecting different levels of MC-LR in spiked real water samples. The recoveries ranged from 86.2 to 118.5% and the relative standard deviation (RSD) was 8.4 to 17.6%. This work provides new inspiration for the construction of effective signal amplification platforms and demonstrates a simple and user-friendly colorimetric method for determination of trace MC-LR.

**Graphical Abstract:**

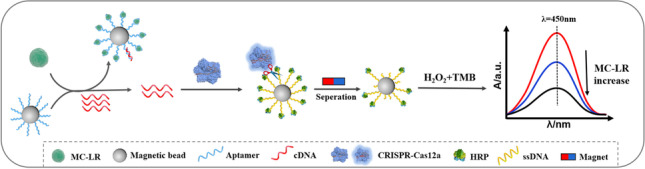

**Supplementary Information:**

The online version contains supplementary material available at 10.1007/s00604-023-05887-9.

## Introduction

Microcystins (MCs), a class of harmful metabolites released from cyanobacteria during freshwater eutrophication, have attracted increasing attention due to their health damage to aquatic organisms and human beings [1]. Specifically, microcystin-LR (MC-LR) exhibits the highest degree of toxicity and prevalence among the more than 270 identified MC variants [2]. MC-LR not only inhibits the activities of type 1 and type 2A protein phosphatases to cause liver failure and tumor exacerbation [3] but also shows high toxicities to the urinary [4], reproductive [5], digestive [6], immune [7], and nervous systems [8]. More seriously, MC-LR can accumulate in aquatic organisms and be transferred into the human body through bioaccumulation [9]. Consequently, even low-level MC-LR in water environment can be a huge threat to human health. The upper limit concentration of MC-LR in drinking water is recommended to be 1 ng·mL^−1^ by the World Health Organization (WHO) [10]. Therefore, the need for a rapid and sensitive analysis technique for trace-level MC-LR detection has become pressing.

Traditional methods for detecting MC-LR, such as high-performance liquid chromatography (HPLC) and liquid chromatography-tandem mass spectrometry (LC–MS), possess certain drawbacks, including the necessity for sophisticated instruments and complicated operation processes [11, 12]. On the contrary, modern optical-based analysis methods, especially colorimetric methods, have higher potential application value due to the use of simpler equipment, lower cost, and easier operation [13, 14]. For example, Lei et al. used MC-LR aptamer to control the peroxidase-mimicking activity of Ti_3_C_2_NSs to develop a label-free colorimetric aptasensor for MC-LR [15]. Owing to the potential of visual signal detection, colorimetric assays can be deployed in resource-limited settings [16]. However, colorimetric assays have inherent shortcomings of low detection sensitivity. The limit of detection (LOD) of colorimetric assays is generally in the level of nanograms or micrograms per milliliter [17, 18]. To achieve sensitive detection with colorimetric assays, the most effective way is to integrate an appropriate signal amplification into the analytical system.

The CRISPR-Cas system, composed of clustered regularly interspaced short palindromic repeats (CRISPR) and CRISPR-associated (Cas) enzymes, has been explored to develop different diagnostic tools in recent years [19, 20]. Some Cas enzymes can selectively recognize DNA sequences and be activated to indiscriminately cleave DNA reporters [21–23], endowing the CRISPR-Cas system with the capability of signal amplification. Thanks to the successful selection of a specific DNA aptamer with a considerable affinity for MC-LR (*K*_d_ = 50 ± 12 nM) by Zourob’s group [24], the combination of aptamer-target recognition with CRISPR-Cas system to develop sensitive analytical assays for MC-LR has become possible. In a previous work, we developed a CRISPR-Cas14a-based fluorescence assay for the quantitative analysis of MC-LR. The activation of CRISPR-Cas14a occurred through a complementary DNA (cDNA) derived from the MC-LR aptamer. The interaction between MC-LR and aptamer leads to the release of cDNA to activate CRISPR-Cas14a. The subsequent Cas14a-catalyzed nuclease reaction generated a fluorescence signal. The analytical method was applied for the quantification of MC-LR with a linear range of 50 pg·mL^−1^ to 1 µg·mL^−1^ [25]. In another study, Kang et al. used CRISPR-Cas12a as a signal amplification tool to establish an on-site platform for MC-LR determination. The working principle was also based on MC-LR–induced release of cDNA from MC-LR aptamer, and the released cDNA was used to activate CRISPR-Cas12a. Benefiting from the CRISPR-Cas12a amplification, the authors were able to detect MC-LR with LOD of ~ 3 fg·mL^−1^ using a fluorescence measurement, and LOD of 1 pg·mL^−1^ in a lateral flow assay [26]. These two studies verified the efficacy of utilizing the CRISPR-Cas system as an amplification tool in developing sensitive detection methods for MC-LR. Thus, it becomes reasonable that the sensitivity of colorimetric assay can be greatly improved through the integration of CRISPR-Cas into the analytical system.

To translate the CRISPR-Cas cleavage event into a colorimetric signal, current research mainly focused on gold nanoparticles (Au NPs) for signal readout. The Au NPs usually acted as the final signal generator but did not contribute to amplifying the signal [27–30]. Horseradish peroxidase (HRP), a widely applied natural enzyme in enzyme-linked immunosorbent assay (ELISA), could promote the conversion of 3,3′,5,5′-tetramethylbenzidine (TMB, colorless) into oxidized TMB (oxTMB, blue). The resultant oxTMB has a characteristic absorption peak and can be easily quantified [31, 32]. Therefore, HRP can translate biological recognition events, such as antigen–antibody recognition, into colorimetric signals. More importantly, HRP itself can amplify molecular recognition signal by catalyzing colorimetric reactions [33].

This work aimed to develop a dual-amplification system by coupling the trans-cleavage activity of CRISPR-Cas12a with HRP-catalyzed colorimetric reaction to detect MC-LR. HRP is immobilized on magnetic beads through a short ssDNA linker (MB-ssDNA-HRP). The cDNA of the MC-LR aptamer was designed as an activator of Cas12a. The binding of MC-LR leads to the release of the cDNA, which in turn activates Cas12a to cleave the ssDNA linker, resulting in the dissociation of HRP from the MBs. Then, the residual HRP on the magnetic beads (MB-ssDNA-HRP) catalyzes the conversion of the TMB-H_2_O_2_ substrate to generate a readable color signal. The colorimetric assay based on HRP and CRISPR-Cas system provides a portable and cost-effective system for MC-LR detection.

## Experimental section

### Reagents and apparatus

EnGen Lba Cas12a and 10 × NEB buffer 2.1 were obtained from New England Biolabs (USA). Dynabeads™ M-280 streptavidin (MBs) was procured from Thermo Fisher Scientific Co., Ltd. (USA). Microcystins, including microcystin-YR (MC-YR) and microcystin-RR (MC-RR), were provided by ENZO Life Science (Germany). Ochratoxin A (OTA) was obtained from AH Diagnostics AB (Sweden). MC-LR, HRP, TMB, sodium periodate (NaIO_4_), sodium borohydride (NaBH_4_), ethylene glycol (EG), and hydrogen peroxide (H_2_O_2_) were purchased from Sigma-Aldrich (Sweden). All DNA oligonucleotides, the details of which are provided in Table 1, were acquired from Sangon Biotech. Co., Ltd. (China). crRNA was supplied by GenScript Biotech. Co., Ltd. (Sweden). RNase-free water was used in CRISPR-Cas cleavage experiments, whereas ultrapure water (resistance ≥ 18.2 MΩ·cm) was used in all the other experimental procedures.

**Table 1:** Nucleic acid sequences used in this study

Name	Sequence (5′ → 3′)
MC-LR aptamer	GGCGCCAAACAGGACCACCATGACAATTACCCATACCACCTCATTATGCCCCATCTCCGC
crRNA	UAAUUUCUACUAAGUGUAGAUCCACCUCAUUAUGCCCCAUC
cDNA (16 nt)	AAAAAGATGGGGCATAATGA
cDNA (20 nt)	GATGGGGCATAATGAGGTGG
ssDNA	Biotin-TTATTTTATTTTATTTTATT-NH_2_
PAGE reporter	TTAACCTTTCTCCATACGCGGAAGTGAGGT
Fluorescence reporter	FAM-TTATT-BHQ1

UV–vis spectrophotometer (Cary 60, Agilent Technologies, USA) and fluorescence spectrophotometer (Cary Eclipse, Agilent Technologies, Germany) were used to record the UV–vis absorption spectra and fluorescence spectra, respectively. A dynamic light scattering instrument (Zetasizer Nano ZS, Malvern Instruments, UK) was employed to measure Zeta potential.

### Preparation of MB-ssDNA-HRP reporter

Preparation of DNA-modified magnetic beads (MB-DNA, including MB-aptamer and MB-ssDNA) is detailed in the “Supplementary information” section. The MB-ssDNA-HRP reporter was prepared following a previously reported method with some modifications [34]. First, 100 µL HRP solution (1 mg·mL^−1^) was combined with 100 µL NaIO_4_ (0.05 mol·L^−1^) and kept at 4 °C for 30 min. After addition of 2 µL EG, the reaction was sustained at the ambient temperature for 30 min. The resulting product mixture was subsequently combined with 100 µL of the prepared MB-ssDNA and subjected to additional incubation for 1 h in the dark. After that, 20 µL freshly prepared NaBH_4_ (0.26 mol·L^−1^) was added and the reaction was sustained at 4 °C for 2 h. Following completion of the reaction, the MB-ssDNA-HRP was collected using magnetic separation and rinsed with water. Finally, the MB-ssDNA-HRP was re-dispersed in 100 µL H_2_O and stored at 4 °C for further use.

### Construction of a dual-amplification system for MC-LR detection

To detect MC-LR with the dual-amplification system, in the beginning, the competitive binding reaction between MC-LR and cDNA for interaction with MC-LR aptamer was performed as follows: 0.5 mg·mL^−1^ MB-aptamer and different concentrations of MC-LR were incubated in 1 × Tris–HCl buffer (50 mmol·L^−1^ Tris–HCl and 150 mmol·L^−1^ NaCl, 2 mmol·L^−1^ MgCl_2_, pH 7.5) at room temperature for 20 min. Then, 90 nmol·L^−1^ cDNA was added, and the incubation was continued for another 20 min at room temperature. Finally, MB-aptamer was removed by magnetic separation, and the supernatant was collected for the activation of CRISPR-Cas12a.

To start the dual-amplification, first, 2.5 µL Cas12a (1 μmol·L^−1^), 8.4 µL crRNA (300 nmol·L^−1^), 5 µL 10 × NEB buffer 2.1, and 34 µL RNase-free water were incubated together at 37 °C for 10 min to form CRISPR-Cas12a. Then, 40 µL of the supernatant (obtained in the previous step) and 20 µL of MB-ssDNA-HRP (10 mg·mL^−1^) were added. After the mixture was incubated at ambient temperature for 30 min, the cleaved MB-ssDNA-HRP was collected by magnetic separation. Next, the collected MB-ssDNA-HRP was mixed with 20 µL H_2_O_2_ (10 mmol·L^−1^) and 10 µL TMB (6 mmol·L^−1^) in 170 µL NaOAc buffer (0.1 mol·L^−1^ sodium acetate, 0.2 mol·L^−1^ acetic acid, pH 5.0) and was reacted at room temperature for 30 min. Finally, 50 µL of H_2_SO_4_ (2 mol·L^−1^) was added to the mixture to terminate the colorimetric reaction. The absorbance spectra (300 ~ 600 nm) of the reaction solution were measured by a UV–vis spectrophotometer.

### Real sample analysis

Tap water and river water were applied to assess the practicability of the proposed dual-amplification colorimetric assay. The tap water was collected from the water supply system in the Chemical Center of Lund University. The river water was collected from the central railway station area in Malmo, Sweden. The samples were fortified with varying concentrations of MC-LR (0.1 ng·mL^−1^, 1.0 ng·mL^−1^, and 10 ng·mL^−1^) and then filtered through a 0.45-µm syringe filter, and then analyzed by the proposed assay directly without further treatment.

### Statistical analysis

All experiments were repeated in triplicate with the values expressed as mean ± standard deviation. SPSS25.0 (SPSS, Chicago, USA) was used for statistical analysis. The significant differences between experimental groups were analyzed using one-way analysis of variance (ANOVA). A *P* value of < 0.05 was considered statistically significant.

## Results and discussion

### Principle of the HRP-mediated CRISPR-Cas12a system

**Scheme 1:**
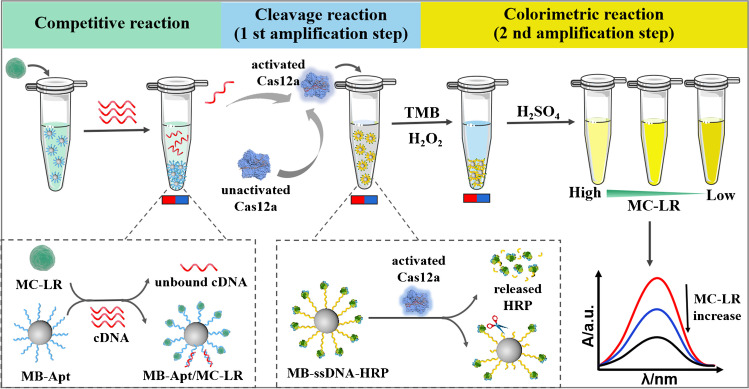
Schematic illustration of the dual-amplification system based on CRISPR-Cas12a and HRP for colorimetric detection of MC-LR

The HRP-mediated CRISPR-Cas12a system for colorimetric detection of MC-LR is executed according to Scheme 1. To enable the implementation of a colorimetric detection, the MB-ssDNA-HRP reporter was first prepared by conjugating HRP to streptavidin-coated magnetic beads utilizing an ssDNA linker. Upon the introduction of MC-LR, the immobilized aptamer located on the magnetic bead (MB-Apt) engaged with MC-LR, inducing a structural alteration in the aptamer that prevented its hybridization with the cDNA. After magnetic separation, the free cDNA, which was designed as a Cas12a activator, triggered CRISPR-Cas12a to trans-cleave the ssDNA linker in the MB-ssDNA-HRP reporter, and thus completing the first signal-amplification step. Upon completion of the magnetic separation to remove the free HRP, the second signal-amplification step started, in which the residual HRP remained on the magnetic beads catalyzed the transformation of the TMB-H_2_O_2_ substrate to alter the color. Consequently, the absorbance intensity was negatively correlated to the MC-LR concentration.

### Characterization of MB-ssDNA-HRP

**Fig. 1:**
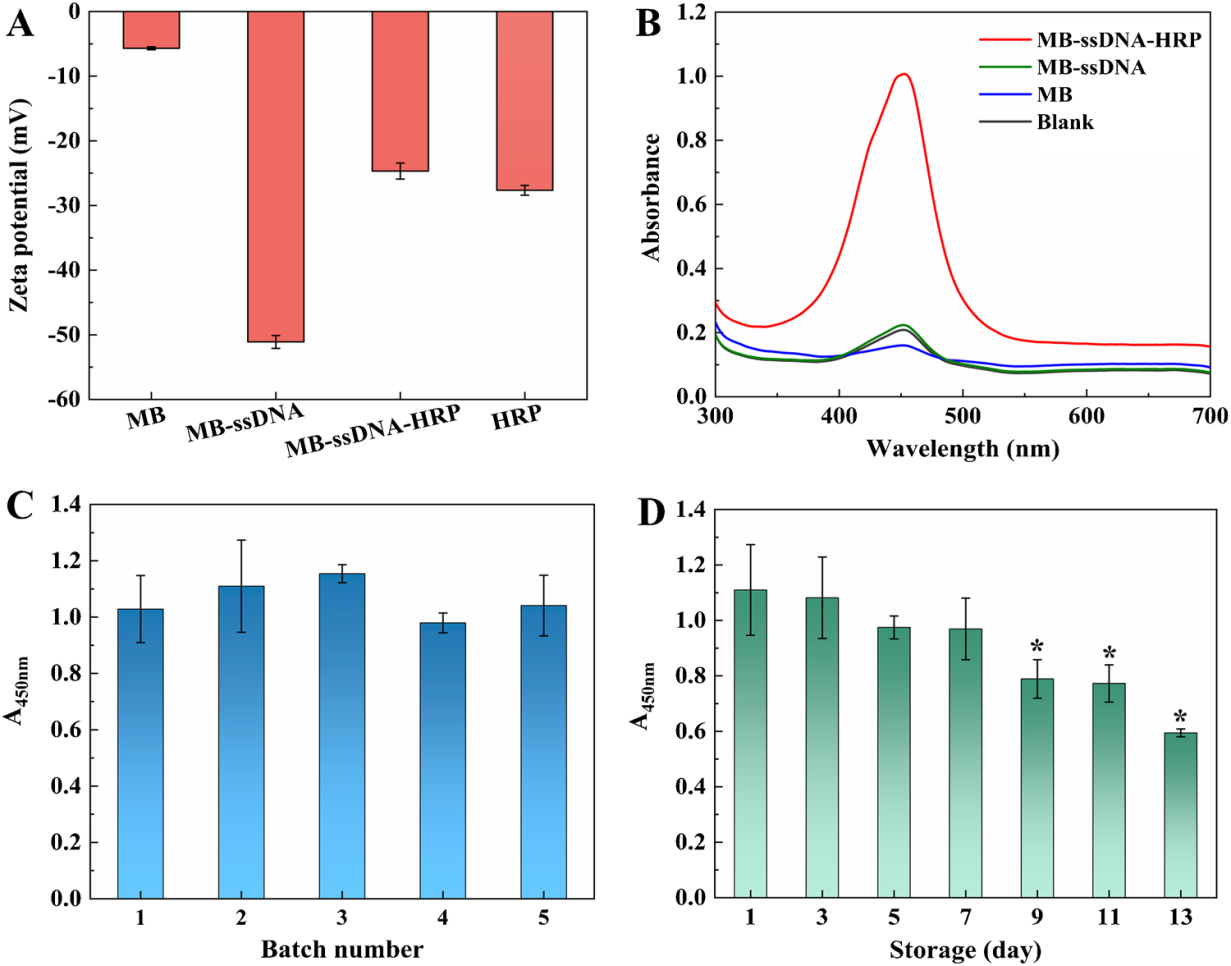
Characterization of MB-ssDNA-HRP. **A** Zeta potential of MB, MB-ssDNA, MB-ssDNA-HRP, and HRP (*C*_sample_ = 1 mg·mL.^−1^). **B** The absorption spectra of end solution from different reaction systems. **C** Comparison of the catalytic capacity of MB-ssDNA-HRP prepared in different batches. **D** The catalytic capacity of MB-ssDNA-HRP measured after different storage times (storage temperature: 4 °C, *P < 0.05 compared with day 1)

As one of the amplification components and signal probes, the MB-ssDNA-HRP plays a key role in the construction of the dual-amplification system. Therefore, the synthesis and properties of MB-ssDNA-HRP were investigated. As presented in Fig. 1A, the Zeta potential showed that the MB was negatively charged (− 5.68 ± 0.22 mV), and the MB-ssDNA carried significantly more negative charge (− 51.10 ± 0.10 mV), indicating that the negatively charged ssDNA was successfully immobilized on the MB. After ligating HRP to MB-ssDNA, the Zeta potential of MB-ssDNA-HRP changed to − 24.67 ± 1.24 mV which was close to the Zeta potential of HRP (− 27.65 ± 0.75 mV). The last Zeta potential change suggests that HRP was successfully conjugated to the surface of MB-ssDNA.

To evaluate the properties of the MB-ssDNA-HRP for practical application, the catalytic activity, repeatability, and stability of the probe were investigated. The catalytic activity of the prepared MB-ssDNA-HRP is shown in Fig. 1B. The MB-ssDNA-HRP was able to catalyze the conversion of TMB-H_2_O_2_ substrate and generate an obvious absorbance at 450 nm, while the MB and MB-ssDNA generated only very weak background absorbance. This result confirms that the immobilized HRP retained its excellent catalytic activity. The repeatability of MB-ssDNA-HRP is presented in Fig. 1C. All the MB-ssDNA-HRP prepared in five different batches could catalyze the conversion of TMB-H_2_O_2_ solution to produce strong absorbance. The ANOVA results showed that there was no significant difference in the catalytic capacity among the different batches (*P* > 0.05). Therefore, the preparation of the probe has satisfactory repeatability. The storage stability was evaluated and the result is presented in Fig. 1D. The relative catalytic capacity of MB-ssDNA-HRP remained stable in the first 7 days (*P* < 0.05), and then gradually decreased (*P* > 0.05). The relatively short storage stability is due to the enzyme’s inherent shortcoming in terms of instability. This limitation may be resolved if a more robust nanozyme is used instead, a subject that we will investigate in future studies.

### Feasibility verification

**Fig. 2:**
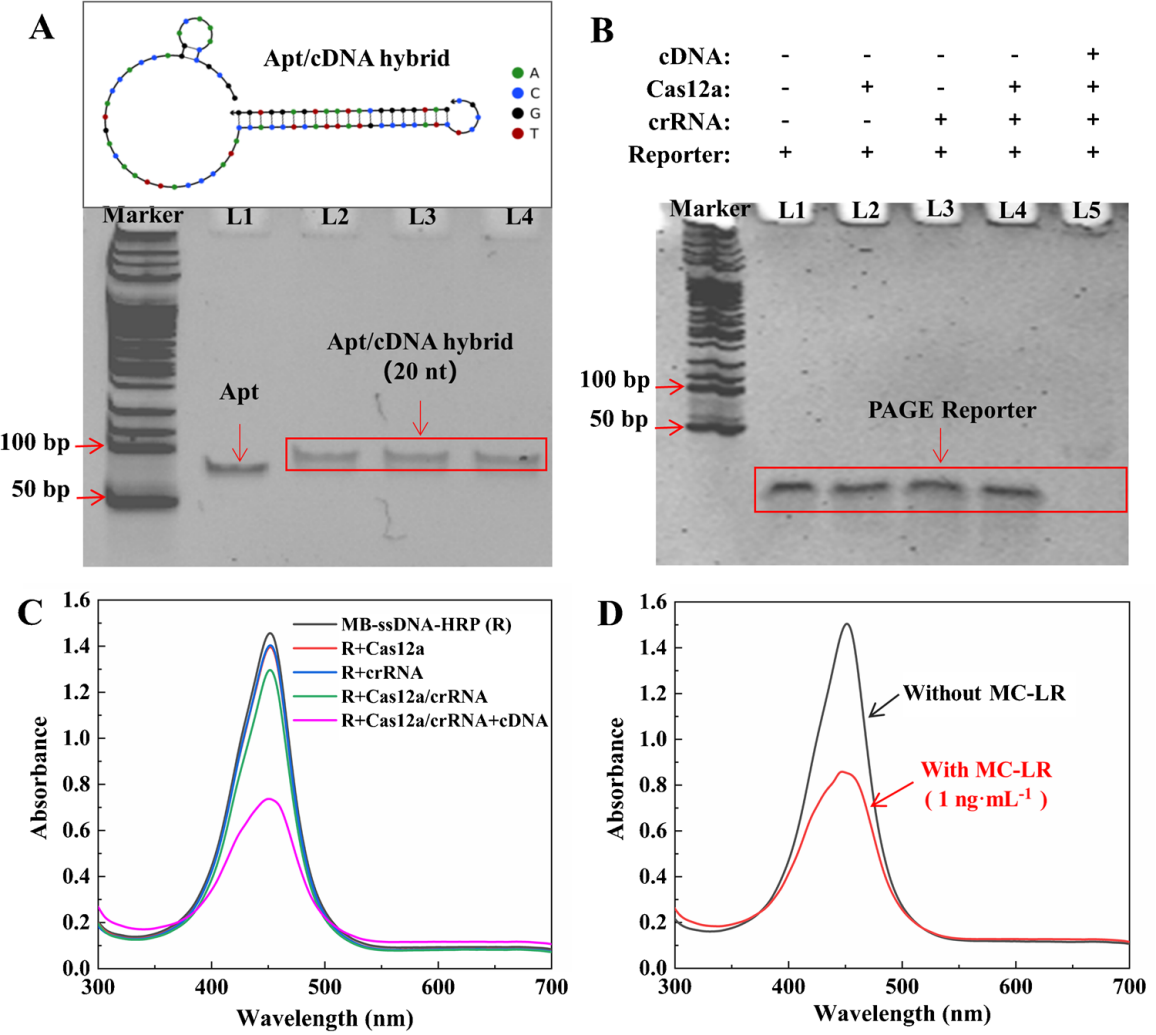
Feasibility of the HRP-mediated CRISPR-Cas12a dual-amplification system for the detection of microcystin-LR. **A** 12% native PAGE analysis of the hybridization between MC-LR aptamer and cDNA (20 nt). **B** Native PAGE analysis of the trans-cleavage ability of Cas12a after activation by cDNA. **C** Absorbance spectra of the products of the colorimetric reactions. **D** Absorbance spectra of the products of the colorimetric reactions in the presence or absence of MC-LR

An appropriate cDNA chain is crucial for MC-LR detection because the hybridization affinity between aptamer and cDNA has a great influence on the binding efficiency of MC-LR with its aptamer. Therefore, two cDNA strands with lengths of 16 nt and 20 nt were synthesized and tested. Both cDNA strands were complementary to the 3′ end of the aptamer. The hybridization ability of the two cDNAs was investigated by native PAGE. As shown in Fig. 2A and Fig. S1, the 20-nt cDNA formed a stable double-stranded complex with the aptamer, while the 16-nt cDNA did not effectively hybridize with the aptamer. Therefore, the 20-nt cDNA (hereinafter referred to as cDNA) was more suitable for MC-LR detection.

The ability of cDNA to activate Cas12a was also examined by native PAGE. As can be seen from Fig. 2B, the intact reporter showed an obvious band near the bottom of the gel (L1). This band did not change after the reporter was treated separately with Cas12a (L2), crRNA (L3), or Cas12a/crRNA (L4). However, after treating the reporter with a mixture of Cas12a/crRNA and cDNA, the reporter band disappeared completely (L5). This result indicates the ability of the cDNA to activate the trans-cleavage property of CRISPR-Cas12a. To further substantiate the activation process of CRISPR-Cas12a by the cDNA, a fluorescence assessment was additionally conducted. Similar to the PAGE result, fluorescence emission was observed only when the fluorogenic substrate was exposed to CRISPR-Cas12a in the presence of the cDNA (Fig. S2).

Next, MB-ssDNA-HRP was first treated with different combinations of Cas12a, crRNA, Cas12a/crRNA, and cDNA and collected by magnetic separation. The collected MB-ssDNA-HRP was then used to catalyze the colorimetric reaction between TMB and H_2_O_2_. The absorbance of different experimental groups is exhibited in Fig. 2C. The untreated MB-ssDNA-HRP produced the highest absorbance. From the MB-ssDNA-HRP that was separately treated with Cas12a, crRNA, or Cas12a/crRNA, the absorbance of the colorimetric reaction decreased only slightly, possibly due to the interference of the added reagents. When MB-ssDNA-HRP was treated with Cas12a/crRNA in the presence of cDNA, the absorbance of the final colorimetric reaction decreased significantly, indicating that HRP has been removed from the magnetic beads due to the cleavage of the ssDNA linker by the activated CRISPR-Cas12a. Despite the relatively large sizes of MB and HRP, it is noteworthy that the ssDNA linker can be effectively cleaved by cDNA-activated Cas12a.

Finally, the feasibility of using the dual-amplification system to detect MC-LR was verified. Figure 2D shows that with the addition of MC-LR, the absorbance intensity of the final colorimetric solution decreased to half of that without MC-LR, even though only 1 ng·mL^−1^ MC-LR was added. This result signifies the HRP-mediated CRISPR-Cas12a system can be applied for facile colorimetric detection of MC-LR.

### Detection of MC-LR

**Fig. 3:**
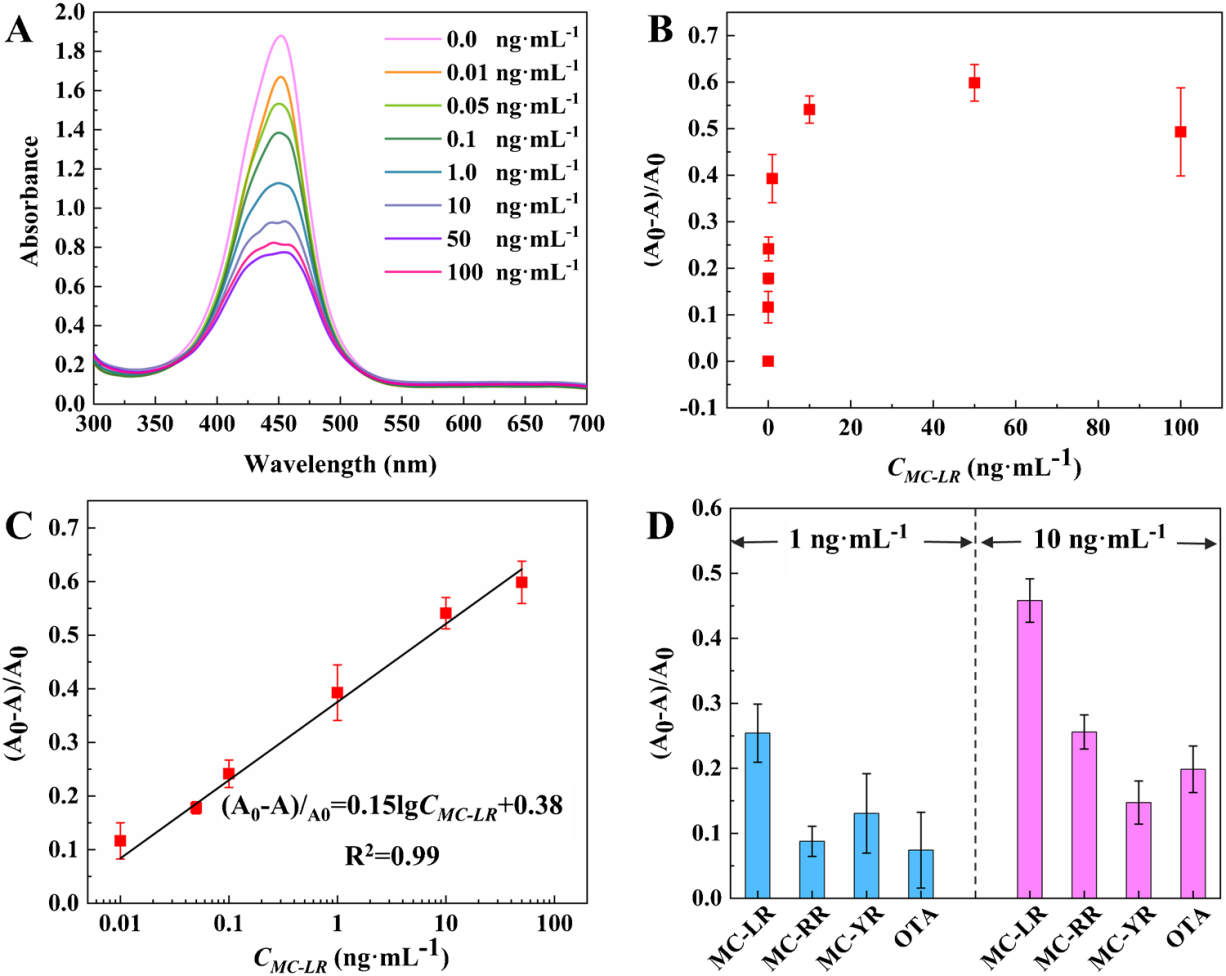
The detection performance of the proposed system for the detection of MC-LR. **A** Absorbance spectra of the colorimetric reaction product caused by different concentrations of MC-LR. **B** The dose–response curve of the proposed system. **C** Relationship between (*A*_0_ − *A*)/*A*_0_ and *C*_MC-LR_. **D** Specificity of the proposed system for the detection of MC-LR

Before evaluating the analytical performance of the dual-amplification approach for the detection of MC-LR, the important experimental conditions including the Cas12a/crRNA concentration, MB-ssDNA-HRP reporter concentration, volume of competition solution, and cleavage time were optimized as 50 nmol·L^−1^, 0.2 mg·mL^−1^, 40 µL, and 30 min, respectively (Fig. S3). Then, the comprehensive evaluation of the analytical performance was conducted (Fig. 3). Figure 3A showed that the absorbance intensity at 450 nm decreased with the increase of MC-LR concentration. This is because more MC-LR present in the sample, more cDNA is unable to hybridize with the aptamer immobilized on the magnetic beads, and the increased free cDNA becomes available to activate the CRISPR-Cas12a, leading to the removal of HRP from the MB-ssDNA-HRP particles. The colorimetric reaction catalyzed by the remaining HRP on the magnetic beads therefore produced a decreased absorbance signal. To normalize the analytical results, the detection signal was defined as (*A*_0_ − *A*)/*A*_0_, which is positively correlated to the MC-LR concentration (Fig. 3B). A favorable correlation was established between the detection signal (expressed as (*A*_0_ − *A*)/*A*_0_) and the logarithm value of MC-LR concentration (Log*C*_MC-LR_) with the range spanning from 0.01 to 50 ng·mL^−1^. Linear curve fitting of the data gave an equation of (*A*_0_ − *A*)/*A*_0_ = 0.15lg*C*_MC-LR_ + 0.38 (Fig. 3C). According to the 3*σ* rule, the LOD was determined to be 4.53 pg·mL^−1^. For comparison, a CRISPR-Cas12a–based fluorescence method was carried out for the detection of MC-LR, in which only the CRISPR-Cas12a was used as an amplification tool, and a fluorogenic reporter was used for signal output (Fig. S4). The results show that the fluorescence signal was linearly correlated to the MC-LR concentration in the range of 0.01–50 ng·mL^−1^ with an LOD of 0.39 pg·mL^−1^ (Fig. S5). Even though fluorescence possesses an intrinsic superiority in sensitivity, leading to a lower LOD in the CRISPR-Cas12a–based fluorescence method, our HRP-mediated CRISPR-Cas12a colorimetric system exhibited a wider linear range and lower LOD than the previously reported colorimetric methods (Table S1). This strongly implies that the developed method is viable for detecting MC-LR with high sensitivity through colorimetry.

To assess the specificity of the dual-amplification system for MC-LR detection, we used MC-RR, MC-YR, and OTA as controls. As shown in Fig. 3D, the strongest detection signal was always observed from MC-LR when tested at two different concentration levels (1 ng·mL^−1^ and 10 ng·mL^−1^), and the detection signal from all the controls was much weaker. All these outcomes indicate that the HRP-mediated CRISPR-Cas12a system possesses the potential to detect MC-LR in a selective manner.

### Real sample analysis

To validate the reliability and applicability of using the HRP-mediated CRISPR-Cas12a system for the purpose of detecting MC-LR in actual samples, standard recovery experiments were carried out in tap water and river water that had been fortified with varying concentrations of MC-LR, including low (0.1 ng·mL^−1^), medium (1.0 ng·mL^−1^), and high (10 ng·mL^−1^) dosages. The results in Table 2 indicate the effective detection of MC-LR in spiked water samples using the dual-amplification colorimetric assay, with an average recovery rate ranging from 86.2 to 118.5%. These results demonstrate that the assay exhibits satisfactory reliability and is highly applicable for practical use.

**Table 2:** Detection of MC-LR in real samples using the HRP-mediated CRISPR-Cas12a system (*n* = 3)

Sample	Spiked (ng·mL^−1^)	Detected (ng·mL^−1^)	Recovery (%)	RSD (%)
Tap water	-	-	-	-
	0.1	0.09	86.2	8.4
	1.0	1.13	113.0	14.6
	10	11.25	112.5	11.3
River water	-	-	-	-
	0.1	0.10	96.3	12.0
	1.0	1.18	118.5	17.6
	10	9.10	91.0	16.4

## Conclusion

In this work, we developed an HRP-mediated CRISPR-Cas12a dual-amplification system for the colorimetric detection of MC-LR. The integration of CRISPR-Cas12a cleavage activity and HRP catalytic ability made it possible to achieve dual-signal amplification and signal conversion, leading to unambiguous absorbance intensity change that can be easily measured with a simple spectrophotometer. With the introduction of a homogeneous competitive reaction between cDNA and MC-LR to bind the specific aptamer, the developed analytical method can accurately and selectively detect low-level concentrations of MC-LR. This HRP-mediated CRISPR-Cas12a dual-amplification system exhibited higher LOD than the CRISPR-Cas12a–based fluorescence method, but the sensitivity and detection range have been greatly improved compared to previously reported colorimetric assays. The dual-amplification system based on CRISPR-Cas12a and HRP exhibits simplicity and convenience in operation and holds considerable promise for high-throughput analysis utilizing microplates. Although the prepared MB-ssDNA-HRP showed limitation in relatively short storage stability, we posit that the implementation of robust nanozymes has the potential to significantly improve the sensitivity and stability of the enzyme-mediated CRISPR-Cas12a dual-amplification system in future research.

## Supplementary Information

Below is the link to the electronic supplementary material.

Supplementary file1 (PDF 365 KB)

## Data Availability

The data generated and analyzed during this study are included in this published article and its supplementary information files.
