# Supplementary material for: Dual-amplification system based on CRISPR-Cas12a and horseradish peroxidase-tethered magnetic microspheres for colorimetric detection of microcystin-LR
Source: Mikrochim Acta. 2023 Jul 20;190(8):314. doi: 10.1007/s00604-023-05887-9 (PMC10359370; doi:10.1007/s00604-023-05887-9)
Supplement: Supplementary file 1 — Supplementary file1 (PDF 365 KB) [file 604_2023_5887_MOESM1_ESM.pdf]

Supporting Information

Dual-amplification system based on CRISPR-Cas12a and horseradish peroxidase-tethered magnetic microspheres for colorimetric detection of microcystin-LR

Pian Wu[a,b], Man Zhang[a], Xiaoting Xue[a], Ping Ding[b*], Lei Ye[a*]

[a] *Division of Pure and Applied Biochemistry, Department of Chemistry, Lund University, 22100 Lund, Sweden*

[b] *Xiang Ya School of Public Health, Central South University, Changsha, Hunan, 410078, China*

[*]Corresponding authors:

E-mail address: lei.ye@tbiokem.lth.se (L, Ye); pingshui@csu.edu.cn (P, Ding).

1. Experiments

1.1 Preparation of DNA-modified magnetic beads (MB-DNA)

The MB-DNA, including MB-aptamer and MB-ssDNA, were prepared using the same procedure. Briefly, 100 μL of MBs (10 mg·mL^{-1}) was washed with 1× B&W buffer (2 mmol·L^{-1} Tris-HCl, 1 mol·L^{-1} NaCl, 0.5 mmol·L^{-1} EDTA, pH 7.5) twice, and was re-suspended in 200 μL 1× B&W buffer. Then, 200 μL biotin-modified aptamer (2 μmol·L^{-1}) or biotin-modified ssDNA (2 μmol·L^{-1}) was added. The mixture was reacted in a rotator under ambient conditions for a duration of 15 minutes. Subsequently, the MB-DNA complex was subjected to serial washes with 1×B&W buffer and water to thoroughly remove excess DNA. Finally, the obtained MB-aptamer was re-dispersed in 200 μL water, while the MB-ssDNA was re-dispersed in 100 μL CBS buffer (0.2 mol·L^{-1} NaH_2PO_4, 0.2 mol·L^{-1} Na_2HPO_4, pH 9.5).

2. Supporting Figures S1-S5

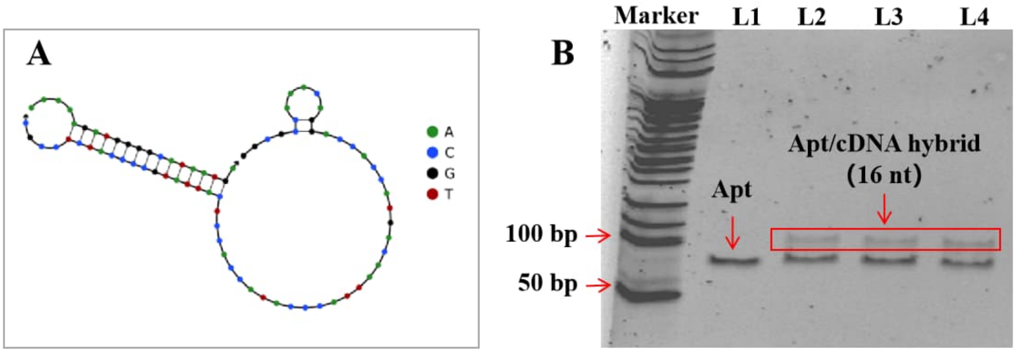

Fig. S1. (A) Scheme of the hybridization between MC-LR aptamer and cDNA (16 nt). (B) 12% native PAGE analysis of the hybridization between MC-LR aptamer and cDNA (16 nt).

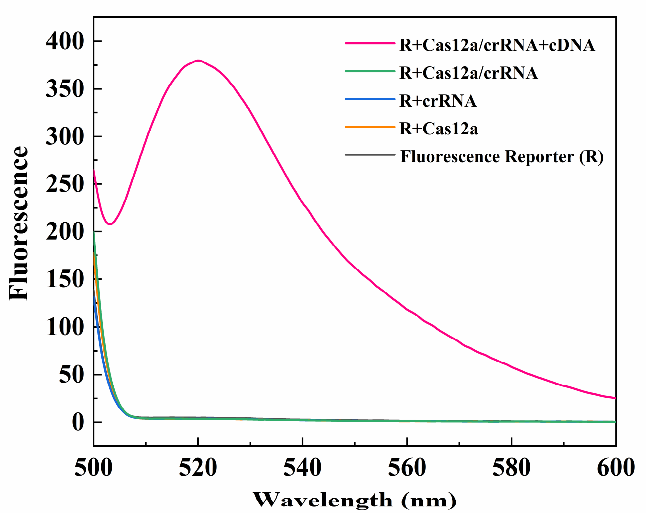

Fig. S2. Fluorescence spectra of the fluorescence reporter in different reaction systems (Ex: 492 nm, Em: 500~600 nm).

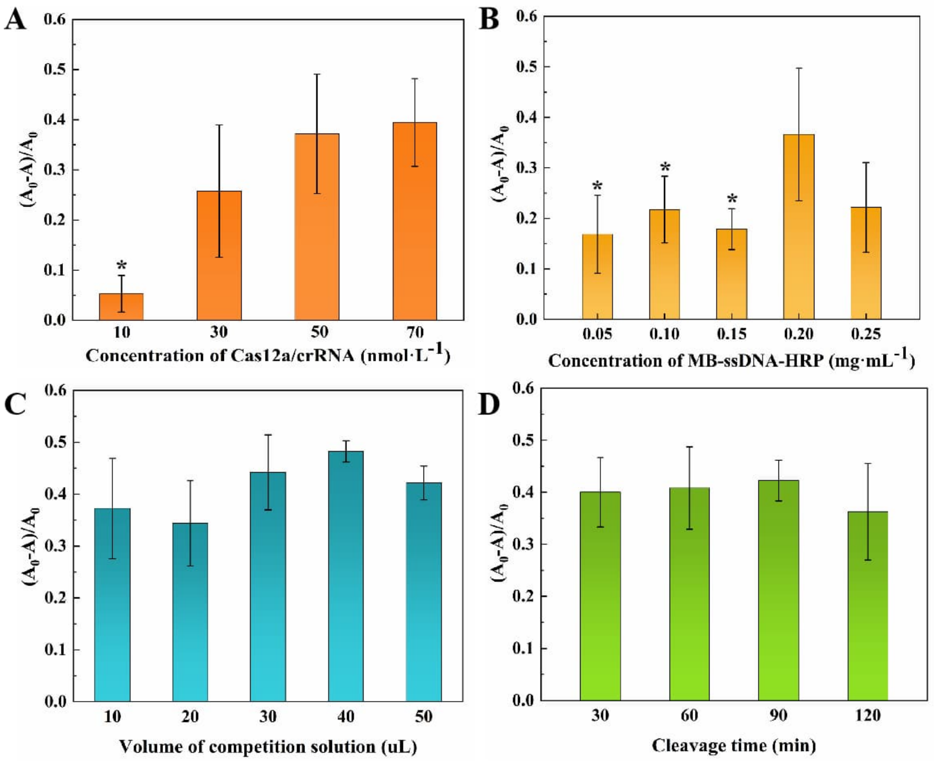

Fig. S3. Optimization of the dual-amplification assay conditions. (A) Effect of Cas12a/crRNA concentration ($^{*}P < 0.05$ compared with 70 nmol·L^{-1}). (B) Effect of MB-ssDNA-HRP concentration ($^{*}P < 0.05$ compared with 0.2 mg·mL^{-1}). (C) Effect of the volume of the competition solution. (D) Effect of cleavage time.

Fig. S3A showed that the Cas12a/crRNA concentration significantly affected the detection signal when the Cas12a/crRNA concentration was lower than 50 nmol·L^{-1}. Above 50 nmol·L^{-1}, further increasing the Cas12a/crRNA concentration caused only negligible increase of the detection signal. Althogh there was no statistically significant difference in the signal generation among 30 nmol·L^{-1}, 50 nmol·L^{-1} and 70 nmol·L^{-1} groups ($P > 0.05$), considering that the signal of 30 nmol·L^{-1} group was significantly lower than the 50 nmol·L^{-1} group, thus, 50 nmol·L^{-1} Cas12a/crRNA was utilized in further experiments.

MB-ssDNA-HRP reporter in the second amplification step acted as a signal transducer. It was a key component in the whole analytical process. Thus, the effect of the MB-ssDNA-HRP reporter concentration on the detection signal was assessed (Fig. S3B). Compared with the signal of 0.2 mg·mL^{-1} group, there was a statistical difference among the 0.05 mg·mL^{-1}, 0.1

$mg \cdot mL^{-1}$, and 0.15 $mg \cdot mL^{-1}$ groups ($P < 0.05$), while the difference between the 0.25 $mg \cdot mL^{-1}$ group was not statistically significant ($P > 0.05$). Therfore, 0.2 $mg \cdot mL^{-1}$ was chosen as the optimal concentration for MB-ssDNA-HRP.

Since a competitive reaction was carried out in the detection process, the volume of the competition solution was also optimized. As shown in Fig. S3C, no statistical difference was found among different volumes of competitive solutions ($P > 0.05$), indicating that the volume of competitive solutions had a small impact on the detection performance. In order to fix variables, 40 μL of competition solution was used for the subsequent experiments.

Finally, we studied the reaction time for activated CRISPR-Cas12a to cleave the MB-ssDNA-HRP reporter. As shown in Fig. S3D, a high detection signal was observed with a cleavage time of 30 min. Further prolonging the cleavage time did not enhance the detection signal ($P > 0.05$), suggesting that 30 min was sufficient for CRISPR-Cas12a to completely cleave the ssDNA linker in MB-ssDNA-HRP.

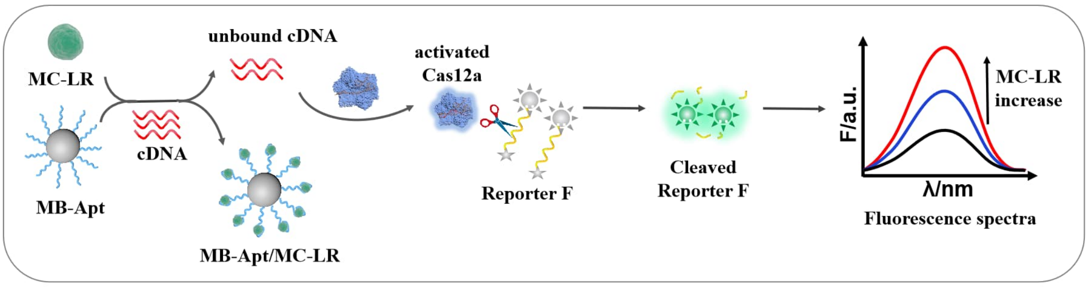

Fig. S4. Schematic illustration of CRISPR-Cas12a-based fluorescence system for the detection of MC-LR.

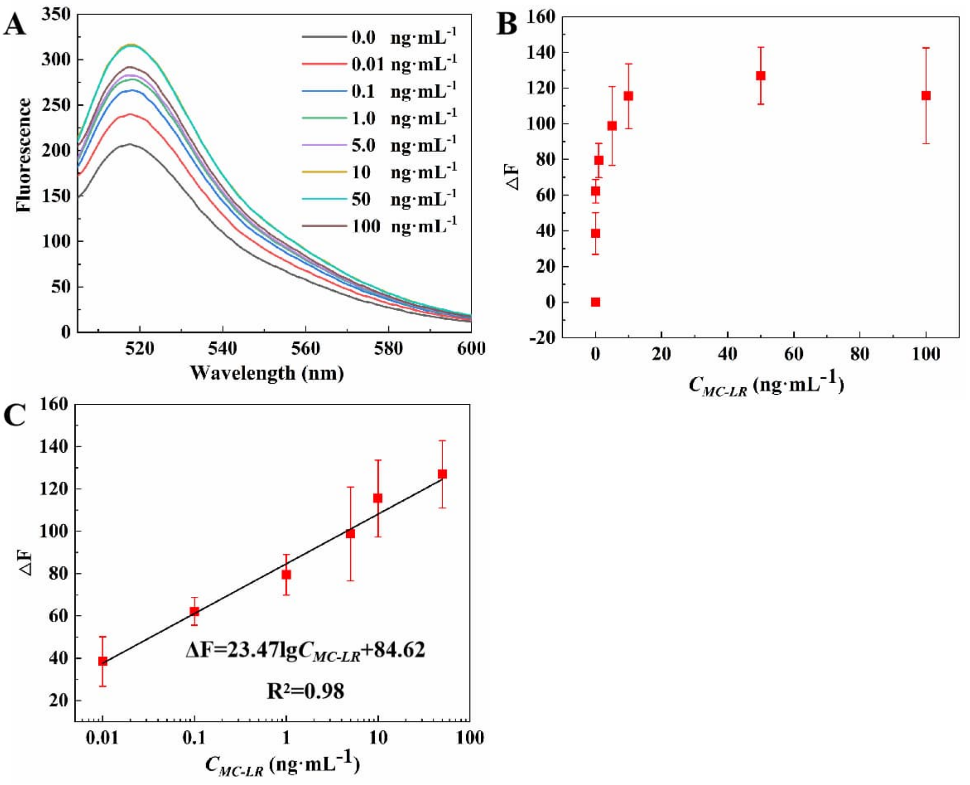

Fig. S5. Detection performance of the CRISPR-Cas12a-based fluorescence system for the detection of MC-LR. (A) Fluorescence spectra resulted from different concentrations of MC-LR. (B) Does-response curve of the fluorescence assay. (C) Relationship between ΔF and $C_{MC\text{-}LR}$.

3. Supporting Tables S1

Table S1. Comparison of the developed assay with reported methods in the literature for the detection of MC-LR.

Detection methods	Signal output	Linear range ($ng \cdot mL^{-1}$)	LODs ($pg \cdot mL^{-1}$)	Refs.
G-quadruplex DNAzyme-based immunosensor	Colorimetry	0.1-10	50	1
Molecularly-imprinted polymers and AuNPs	Colorimetry	0.1-100	40	2
Au-based aptamer sensor	Colorimetry	~0.5-75000	~370	3
Au NPs dimers-based aptamer sensor	Colorimetry	0.1-250	50	4
Surface copper nanoparticles of polydopamine nanosphere	Colorimetry	0.05-25	50	5
graphene oxide hydrogel matrix	Colorimetry	0.65-1000	217	6
PP2A enzyme-entrapping agarose gels	Colorimetry	0.78-3.125	170	7
Ti_3C_2 nanosheets	Colorimetry	0.01-60	6.5	8
CRISPR-Cas12a-based fluorescence method	Fluorescence	0.01-50	0.39	This study
HRP-mediated CRISPR-Cas 12a colorimetric system	Colorimetry	0.01-50	4.53	This study

References

1. Zhu, Y.; Xu, L.; Ma, W.; Chen, W.; Yan, W.; Kuang, H.; Wang, L.; Xu, C., G-quadruplex DNAzyme-based microcystin-LR (toxin) determination by a novel immunosensor. *Biosensors and Bioelectronics* **2011,** *26* (11), 4393-4398.
2. Wu, Z.; He, D.; Cui, B.; Jin, Z., Ultrasensitive detection of microcystin-LR with gold immunochromatographic assay assisted by a molecular imprinting technique. *Food Chemistry* **2019,** *283*, 517-521.
3. Li, X.; Cheng, R.; Shi, H.; Tang, B.; Xiao, H.; Zhao, G., A simple highly sensitive and selective aptamer-based colorimetric sensor for environmental toxins microcystin-LR in water samples. *Journal of Hazardous Materials* **2016,** *304*, 474-480.
4. Wang, F.; Liu, S.; Lin, M.; Chen, X.; Lin, S.; Du, X.; Li, H.; Ye, H.; Qiu, B.; Lin, Z.; Guo, L.; Chen, G., Colorimetric detection of microcystin-LR based on disassembly of orient-aggregated gold nanoparticle dimers. *Biosensors and Bioelectronics* **2015,** *68*, 475-480.
5. Tang, X.; Yin, Z.; Lei, X.; Zeng, Y.; Zhang, Z.; Lu, Y.; Zhou, G.; Li, L.; Wu, X., Colorimetric Method for Sensitive Detection of Microcystin-LR Using Surface Copper Nanoparticles of Polydopamine Nanosphere as Turn-On Probe. *Nanomaterials* **2019,** *9* (3).
6. Abnous, K.; Danesh, N. M.; Ramezani, M.; Taghdisi, S. M., Colorimetric determination of the microcystin leucine-arginine based on the use of a hairpin aptamer, graphene oxide, and Methylene Blue acting as an optical probe. *Microchimica Acta* **2017,** *184* (11), 4451-4457.
7. Sassolas, S.; Catanante, G.; Fournier, D; Marty, J. L., Development of a colorimetric inhibition assay for microcystin-LR detection: Comparison of the sensitivity of different protein phosphatases. *Talanta* **2011**, *85*(5), 2498-2503.
8. Guo, J.; Wang, G.; Zou, J.; Lei, Z., DNA controllable peroxidase-like activity of Ti_3C_2 nanosheets for colorimetric detection of microcystin-LR. *Analytical and Bioanalytical Chemistry* **2023**, *415*, 3559-3569.
